# Plant-Based Bigels for Functional Delivery: Advances in Structural Design and Stabilization Strategies

**DOI:** 10.3390/foods14213699

**Published:** 2025-10-29

**Authors:** Chao Cheng, Xianghui Yan, Dongze Li, Zheling Zeng, Qiangzhong Zhao, Xiujie Zhao, Shaoyun Wang

**Affiliations:** 1 College of Biological Science and Engineering, Fuzhou University, Fuzhou 350108, China; chengchao0812@163.com; 2 College of Food Science and Technology, Nanchang University, Nanchang 330031, China; yanxianghui@ncu.edu.cn (X.Y.); zlzengjx@hotmail.com (Z.Z.); 3 School of Food Science and Engineering, Hainan University, Haikou 570228, China; lidongze@hainanu.edu.cn; 4 School of Food Science and Engineering, South China University of Technology, Guangzhou 510640, China; qzzhao@scut.edu.cn

**Keywords:** plant-based bigels, functional delivery, structural design, stabilization strategies, bioactive compounds

## Abstract

As the increasing demand for clean-label, plant-based, and functional food systems, bigels, an innovative biphasic structured system composed of both hydrogels and oleogels, have emerged as promising research focus for delivering functional ingredients in the food, pharmaceutical, and cosmetic fields. Plant-based bigels, formulated from edible biopolymers and vegetable oils, represent a sustainable and regulatory-compliant delivery platform. This review critically reviews the recent advances in the structural design and stabilization of plant-based bigels, with an emphasis on the regulation of phase behavior and interfacial interactions. Advanced strategies, including stimuli-responsive gelation, Pickering interfaces, and semi-interpenetrating networks, are explored to improve stability and enable targeted gastrointestinal release. Applications in the delivery of polyphenols, omega-3 fatty acids, lipophilic vitamins, and probiotics are highlighted, underscoring the relationship between structural construction and delivery performance. Furthermore, current challenges and potential solutions concerning stability enhancement, bioavailability improvement, and industrial scalability are outlined. Future research directions are proposed to address existing gaps and to further exploit the potential of plant-based bigels for functional compound delivery.

## 1. Introduction

In recent years, there has been a sustained transition in the global food industry toward plant-based, clean-label, and nutritionally enriched products. Within complex food matrices, the effective encapsulation and predictable release of food bioactive compounds, such as polyphenols, carotenoids, vitamins, probiotics, and ω-3 polyunsaturated fatty acids, have emerged as a key challenge in the structural design and functional delivery systems [1,2]. Bigels, defined as biphasic systems comprising a hydrogel and an oleogel phase, typically form interpenetrating networks, bicontinuous structures, or dispersed-phase morphologies (O/H or H/O), and were first introduced by Almeida et al. in 2008 as pharmaceutical delivery vehicles [3,4]. Recently, they have garnered increasing attention in food science due to their structural versatility, favorable biocompatibility, and improved physical stability. Notably, bigels are capable of simultaneously incorporating both hydrophilic and lipophilic compounds, thereby enabling the co-delivery and sequential release of structurally and functionally diverse bioactives within a single composite matrix [5]. Compared with conventional mono-phase delivery systems, bigels offer enhanced physical stability, superior encapsulation efficiency, and highly tunable viscoelastic and microstructural characteristics [6,7], positioning bigels as promising material platform for food-grade functional delivery applications.

In this review, the term plant-based bigels specifically refers to biphasic hydrogel-oleogel systems in which both gelators and structural components are derived entirely from plant sources. While food-grade bigels and edible bigels are broader categories that may include systems utilizing animal-derived or synthetic gelators approved for ingestion, plant-based bigels emphasize sustainability, vegan compliance, and alignment with the clean-label paradigm in modern food design. For clarity and consistency, the term plant-based bigels is used throughout this manuscript. Compared with animal-derived bigels, plant-based bigels utilize vegetable oils as the lipid phase, structured with agents such as plant waxes, monoglycerides, or phytosterols [8,9,10]. The aqueous phase is typically composed of hydrocolloids including alginate, pectin, gellan gum, xanthan gum, cellulose derivatives, or plant proteins [11,12,13]. This formulation approach offers advantages in terms of ingredient sustainability, regulatory acceptance, and compatibility with plant-based product development. The structural properties of bigels can be modulated through several key variables, including the ratio and continuity of the hydrogel and oleogel phases, the gelation sequence and conditions, and interfacial stabilization strategies [14]. These factors collectively influence the rheological behavior, interfacial stability, and release kinetics of the encapsulated actives. To enhance the performance of plant-based bigels under food-relevant conditions, including mechanical processing, storage, and gastrointestinal digestion, multiple stabilization strategies have been explored. Hydrogel networks can be reinforced via ionic crosslinking, enzymatic treatment, or physical entanglement, while oleogel structuring can be optimized through crystal engineering to control crystal morphology and packing density [15,16,17,18]. Additionally, interfacial functionalization strategies, including synergistic use of emulsifiers with Pickering particles, incorporation of natural antioxidants, and integration of stimuli-responsive elements (e.g., pH-, or thermo-sensitive moieties), have been explored to mitigate phase separation, retard oxidative deterioration, and achieve targeted release [19,20]. Recent studies have demonstrated that cellulose-based bigels can maintain structural integrity under shear [21], and that bigel matrices can improve the survival of encapsulated probiotics and enable site-specific delivery of sensitive compounds such as pigments [22,23].

Although significant progress has been made in the formulation and functional characterization of bigel systems, there remains a need for a more comprehensive understanding of plant-based bigels in terms of their structural design, co-encapsulation capabilities, and the relationship between microstructure and bioavailability. Since previous reviews have extensively discussed the general composition and preparation methods of bigels [1,24,25,26], the current article does not reiterate those foundational aspects. Instead, this review focuses specifically on recent advances in the structural design and stabilization strategies of plant-based bigels, analyzes the relationship between structural construction and delivery performance, and highlights current knowledge gaps and outlines future directions for the integration of plant-based bigels into complex food systems.

## 2. Structural Design and Stabilization Strategies of Plant-Based Bigels

### 2.1. Typical Plant-Based Gel Components

The structural and functional integrity of plant-based bigels fundamentally relies on the crosslinking degree and network strength of the individual gel phases, both of which are, in turn, governed by the nature of gelators. Typically, edible and biocompatible plant-derived biopolymers, including polysaccharides and proteins, are used to construct the hydrogel phase, while the lipophilic phase consists of vegetable oils or structurally modified derivatives. The use of plant-derived biopolymers not only aligns with clean-label formulation and sustainability objectives but also offers a promising alternative to animal-derived gelatins [16].

Plant-derived polysaccharides and proteins serve as primary hydrogel-forming agents due to their abundance, biocompatibility, and tunable gelation mechanisms. Polysaccharides such as alginate [C_6_H_7_O_6_Na]_n_, κ-carrageenan (a sulfated galactan polymer with alternating 3-linked β-D-galactose and 4-linked α-D-galactose-3,6-anhydro units), and pectin [C_6_H_8_O_6_]_n_ form hydrogels through ionic or hydrogen-bond crosslinking [8,27,28]. Cellulose derivatives, including carboxymethyl cellulose (CMC) [C_6_H_7_O_2_(OH)_x_(OCH_2_COONa)_y_]_n_ and hydroxypropyl methylcellulose (HPMC), gel via chain entanglement and hydrophobic association [11,29]. Agar and gellan gum undergo coil-to-helix transitions upon cooling, forming thermo-reversible networks, while xanthan gum and guar gum stabilize aqueous phases through hydrogen-bonded double helices [9,14,30,31]. At the molecular level, polysaccharide-based hydrogels exhibit repeating saccharide units linked via β-(1→4) or α-(1→3) glycosidic bonds, which generate a hierarchical polymeric framework capable of immobilizing large amounts of water. Many of them exhibit thermal reversibility, tunable gelation kinetics, and strong water-holding capacity, which are essential for maintaining moisture retention and matrix integrity in the bigel system.

In addition to polysaccharides, plant proteins are increasingly being utilized as hydrogel-forming agents. Soy and pea proteins can form thermally induced hydrogels under specific pH and ionic strength conditions [32]. Although plant protein hydrogels are generally weaker and less elastic compared to animal gelatin, recent studies have demonstrated that synergistic combinations with polysaccharides, such as pea protein and carboxymethyl cellulose, can produce mechanically robust, biopolymer-rich hydrogels suitable for bigel formation [33]. Plant-based hydrogelators offer a balance of viscoelasticity, edibility, and nutritional beneficial (e.g., dietary fiber content), making them ideal for functional delivery systems. The selection must consider gel strength, gelation temperature, and interactions with other components. For instance, agar and κ-carrageenan produce firm gels at low concentrations but may lack flexibility; adding locust bean gum can improve elasticity via synergistic interactions [34,35,36]. Thus, binary or ternary mixtures of hydrocolloids are commonly employed to tailor the mechanical properties, thermal stability, and microstructure of the hydrogel.

Plant-based oleogelators are generally categorized as low-molecular-weight gelators (LMWGs) and polymeric or high-molecular-weight gelators (HMWGs), differing in assembly mechanism and network structure [37,38]. LMWGs (typically <1 kDa), including monoglycerides (glycerol monostearate, C_21_H_42_O_4_), stearic acid (C_18_H_36_O_2_), phytosterols (β-sitosterol, C_29_H_50_O), and γ-oryzanol (C_40_H_58_O_4_), self-assemble through hydrogen bonding and van der Waals interactions to form crystalline or fibrillar networks that immobilize oil molecules [39,40,41]. Phytosterols and oryzanol (from rice bran) can also pair with fatty acids to form self-assembled fibrils in oil phase, though they often require combination (e.g., β-sitosterol with γ-oryzanol) [42]. Their gelation is highly sensitive to temperature and shear, and is typically triggered by controlled cooling [38]. Effective gelation depends on a delicate balance between gelator molecules and gelator molecule–oil phase interactions, which govern both network morphology and mechanical strength [26]. Natural waxes (beeswax, candelilla, carnauba) consist primarily of long-chain esters (RCOOR′) that crystallize into lamellar arrays upon cooling, offering high gel strength and thermal stability at relatively low concentrations [43,44].

HMWGs, such as ethyl cellulose [C_12_H_23_O_6_]_n_, produce viscoelastic oil networks via polymer chain entanglement and hydrogen bonding. Their gelation involves solvated polymer chains forming a percolating network as temperature decreases, generating flexible and thermo-reversible matrices. Phospholipids (e.g., lecithin, C_42_H_84_NO_9_P) can serve dual roles as emulsifiers and oleogelators, enhancing interfacial compatibility and structural cohesion within bigel matrices.

HMWGs, such as polysaccharides and proteins, form three-dimensional networks through polymer chain entanglement, hydrogen bonding, or physical crosslinking. Compared to LMWGs, HMWGs typically gel oil at lower concentrations (<2%) and form networks with viscoelastic behavior governed by molecular weight, chain conformation, and oil compatibility [1]. A prime example is ethyl cellulose (EC, [C_12_H_23_O_6_]_n_), a chemically modified cellulose that dissolves in hot oil and forms thermo-reversible and flexible gels upon cooling via a percolating polymer network, though typically with lower hardness [42]. Food-grade phospholipids (e.g., lecithin, C_42_H_84_NOP) can serve dual roles as emulsifiers and oleogelators, enhancing interfacial compatibility and structural cohesion within bigel matrices [45].

The molecular design principles of gelators, including chain length, polarity, degree of substitution, and crystallization behavior, directly influence the microstructural organization, phase morphology, and mechanical stability of bigels. Oleogelator selection should be tailored to the desired phase morphology and functional performance of the bigel system. Typically, beeswax-based bigels exhibited a hydrogel-in-oleogel structure with superior gel strength and oil retention, but are prone to phase separation under stress, while glycerol monostearate (GMS) promotes an oleogel-in-hydrogel structure with higher freeze–thaw stability and thermo-reversibility, although with limited structural recovery after thermal cycling [46]. Notably, hybrid oleogel systems combining multiple gelators are increasingly explored to optimize network synergy, improving mechanical robustness, elasticity, and structural stability in complex food matrices.

### 2.2. Regulation of Phase Behavior in Plant-Based Bigels

**Figure 1:**
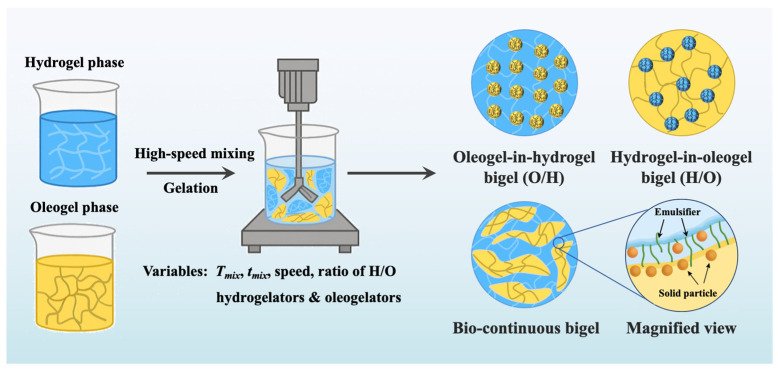
The preparation process of bigels and the structural diagrams of different types.

The phase behavior of plant-based bigels is predominantly governed by the volume fraction of each gel phase (φ), the dispersed or continuous morphology formed during mixing, and the degree of network interpenetration between the hydrogel and oleogel phases. Depending on these factors, bigels typically exhibit oleogel-in-hydrogel (O/H), hydrogel-in-oleogel (H/O), or bicontinuous networks, as schematically illustrated in Figure 1 [14]. Recent research has demonstrated that these morphologies represented a continuous spectrum of multiscale structural architectures, rather than discrete states, where phase continuity and interfacial connectivity jointly determined mechanical integrity, mass-transfer behavior, and storage stability [33,40]. Quantitative structure mapping using confocal laser scanning microscopy (CLSM), cryo-SEM, and X-ray micro-computed tomography (micro-CT) has enabled direct visualization of bicontinuous networks and established clear correlations between microstructural connectivity and viscoelastic behavior.

Precise control over phase proportions, gelation kinetics, and processing variables (e.g., shear rate, temperature, and mixing sequence) is essential for achieving predictable phase inversion and stable interfacial microstructures, with inversion primarily governed by the oleogel-hydrogel ratio [47]. In beeswax oleogel-HPMC hydrogel-based bigels, increasing the oleogel fraction to 55% disrupted the continuity of the hydrogel phase [48]. This transition exhibited as fragmentation of the hydrogel into dispersed domains and partial coalescence of oil droplets into contiguous regions, resulting in a semi-continuous microstructure. At higher oil fraction (≥60%), complete inversion to an H/O structure occurred [48]. This gradual transformation from O/H to bicontinuous and eventually H/O morphologies is accompanied by systematic alterations in rheological properties, an increase in elastic modulus (G′), extension of the linear viscoelastic region (LVR), and decrease in tan δ, which collectively modulate textural firmness, spreadability, and diffusion-controlled release [49]. Hydrogel-continuous bigels generally exhibit higher rigidity and superior retention of hydrophilic compounds, while oil-continuous systems enhance solubilization and protection of lipophilic components. Higher oleogel fractions introduce additional crystalline domains, which contributes to orderly molecular packing and improved microstructural coherence. Differential scanning calorimetry (DSC) and thermogravimetric analysis (TGA) provide insight into crystallization behavior, thermal reversibility, and melting transitions, which influence texture and shelf stability. As demonstrated by Martins et al., increasing oleogel content not only promoted crystalline alignment within the bigel matrix but also strengthened gel network integrity and suppresses coalescence [15]. Such ordered microstructure plays a pivotal role in maintaining phase integrity and enabling controlled release, particularly under thermal or mechanical stress conditions [26].

Achieving a bicontinuous network in bigels requires the simultaneous or cooperative formation of both hydrogel and oleogel networks. Two primary fabrication strategies have been reported: co-gelation and sequential gelation. In the co-gelation approach, a high-temperature emulsification step is followed by controlled cooling, triggering simultaneous gelation in both the aqueous and lipid phases.

This enables the formation of intertwined three-dimensional networks at the oil-water interface, facilitated by reduced interfacial tension during thermal processing [50]. In contrast, sequential gelation involves the pre-formation of gel network, typically the continuous phase, followed by emulsification and subsequent structuring of the second phase, thus promoting interfacial interlocking [2,51]. The essential distinction between these strategies lies in the energy dynamics during gelation. Thermo-setting co-gelation typically results in enhanced network interpenetration and a more homogeneous phase structure, attributable to the gradual energy dissipation and network assembly. By comparison, cold-set bigels exhibit a more ordered network and lower hydrogel porosity, likely due to the slower network formation kinetics and reduced thermal agitation [52]. Effective interpenetration and co-continuity of bicontinuous networks require matched gelation rates and balanced mechanical properties between the two phases; premature solidification of one network can lead to weakening mechanical properties and favoring phase separation [50]. Studies on gellan gum-monoglyceride-based bigels have shown that dispersed hydrogel domains can act as active fillers, reinforcing the composite network [53]. Furthermore, systems containing β-sitosterol and monoglycerides demonstrated rapid diffusion and crystallization at the hydrogel-oleogel phase interface, intensifying interfacial interactions and reinforcing the bigel structure. However, excessive interfacial crystallization may yield a rigid shell, restrict spatial flexibility, and ultimately decrease the elasticity [54]. In contrast, bigels formulated with β-sitosterol and γ-oryzanol displayed slower interfacial diffusion and limited crystallization, resulting in weaker hydrogel-oleogel phase interactions but improved structural flexibility and reduced rigidity. These findings highlight a fundamental balance between network stiffness and elasticity, dictated by the interfacial assembly kinetics and crystallization behavior of the structuring agents.

Processing conditions, including mixing temperature, shear intensity, and shear duration, are critical determinants of the final microstructure, rheological profile, and mechanical integrity of plant-based bigels [26]. Thermal homogenization facilitates fine droplet dispersion and improved interfacial uniformity by reducing the viscosity of both phases and enhancing mixing efficiency. The processing temperature should be maintained above the crystallization onset temperature of the oleogelators to ensure complete melting and homogeneous mixing, followed by controlled cooling to promote uniform nucleation and prevent premature crystallization or crystal coarsening, which would otherwise compromise phase uniformity and weaken network connectivity [26,52]. Conversely, cold-state mixing is advantageous when processing thermo-sensitive bioactives but typically results in broader size distributions and less uniform phase continuity due to limited molecular mobility. Increasing shear rate during mixing can effectively decrease the size of dispersed gel particles, promoting a more homogeneous network. Nevertheless, excessive shear may disrupt partially formed gel structures, compromise network integrity, and diminish viscoelastic strength [15,55]. Therefore, optimizing the temperature-shear interplay is essential to balance gel particle size uniformity and network preservation. Well-designed processing protocols can simultaneously enhance structural coherence, tune viscoelasticity, and preserve the functional performance of encapsulated bioactives, ultimately enabling the rational design of bigels with predictable and reproducible delivery behavior.

### 2.3. Interfacial Design for Plant-Based Bigels

The structural stability of plant-based bigels relies on the interfacial compatibility between the hydrogel and oleogel phases, where a delicate balance between attractive and repulsive forces determines the extent of phase continuity and network integration [56]. Although both gelators possess intrinsic abilities to immobilize water and oil phases, interfacial incompatibility or viscoelastic mismatch may result in premature phase separation during processing or before complete gelation. Targeted interfacial design is therefore essential to control droplet distribution, enhance interfacial connectivity, and confer predictable mechanical and release characteristics.

Incorporation low concentrations of food-grade emulsifiers or particulate stabilizers can effectively reduce interfacial tension and promotes the formation of fine and uniform dispersed domains. Liu et al. demonstrated that combining phytosterol-GMS oleogels with κ-carrageenan hydrogels resulted in ordered “embedded crystal” structures through hydrogen bonding between the C3 hydroxyl of phytosterols and the glycerol head group of GMS [27]. This structural arrangement not only reinforced the network against external stress but also enhanced hydrogen bonding with water molecules at the oil–water interface, reinforcing bigel interfacial stability. Similarly, studies on soy protein isolate (SPI)-based bigels revealed that increasing SPI concentration can induce a phase inversion from H/O to O/H, and subsequently back to H/O, as SPI functions as a natural emulsifier and modulates interfacial proton binding and free water content, highlighting the tunable nature of phase morphology via protein-mediated interfacial stabilization [57].

**Figure 2:**
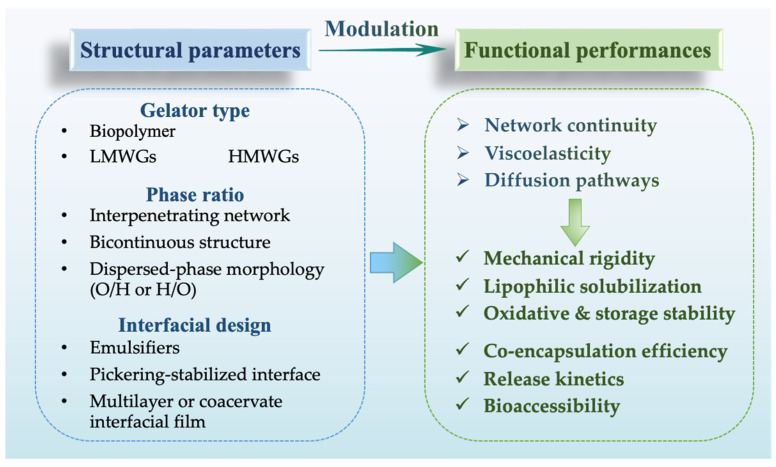
Key structure-function relationships of plant-based bigels.

Certain amphiphilic compounds, such as monoglycerides, diglycerides, and glycerol monostearate, exhibited dual functionality in bigels serving as emulsifiers at the oil-water interface while simultaneously participating in the crystalline structuring of the oleogel phase [26,58]. Lecithin and glycerol (≤5%) have been shown to facilitate the formation of bicontinuous networks in gelatin-monoglyceride bigels, simultaneously enhancing gel strength and phase connectivity [59]. Beyond reducing interfacial energy, these additives influence phase continuity and permeability, enabling the construction of interpenetrating or semi-interpenetrating networks with superior textural and rheological performance. This dual role improves both interfacial stabilization and bulk mechanical properties. Nutter et al. demonstrated that mixed-size crystal domains in monoglyceride-wax oleogels formed densely packed networks, significantly enhancing oil-binding capacity and gel strength [60]. Similarly, Samui et al. constructed in situ bigels composed of glycerol monostearate oleogels and gelatin hydrogels, where the presence of lecithin and glycerol at the interface synergistically promoted bicontinuous network formation and improved phase connectivity and cohesiveness [59]. These studies collectively highlight that precise interfacial modulation, through rationally selecting emulsifiers, co-surfactants, and crystal-structuring agents, can also alter phase continuity, network interpenetration, and diffusion pathways, enabling the construction of advanced semi- or fully interpenetrating networks with enhanced structural elasticity and tailored release kinetics, as illustrated in Figure 2.

A rapidly advancing strategy in bigel design involves using edible particles to construct Pickering-stabilized interface. Food-grade solid particles, such as zein nanoparticles, cellulose nanocrystals (CNCs), and modified starch granules, irreversibly adsorb at the oil-water interface, assembling into viscoelastic interfacial films that suppress coalescence and droplet migration and thereby enhance the bulk network [61,62,63]. Interfacial tension measurements and contact angle analysis are used to characterize the wettability and interfacial anchoring strength of particles, which is a key factor in Pickering stabilization. Zein-stabilized beeswax-oleogel/cassava-starch hydrogel bigels remain stable at oil fractions exceeding 40% without added surfactants, and the densely packed particle shells enable rheological tunability by adjusting the oil phase while maintaining structural integrity [64]. A critical factor in achieving effective Pickering stabilization lies in the wettability and interfacial anchoring strength of the particles, which can be finely tuned through surface modification. Adjusting the hydrophobicity of CNCs has been shown to regulate interfacial elasticity and diffusional resistance, thereby enabling precise control over microstructure development and functional performance in bigels [61]. Wang et al. synthesized amphiphilic nanoparticles between SPI and acylated anthocyanins via a Mannich reaction and incorporated them into beeswax-guar gum bigels, where hydrogen bonding and hydrophobic interactions strengthened the network, while preferential interfacial migration enhanced phase connectivity and stability, yielding a synergistic improvement in both interfacial and bulk properties [65]. Particle-stabilized bigels support clean-label formulation while suppressing coalescence and Ostwald ripening, thus extending shelf life.

A complementary strategy involves leveraging electrostatic assembly to build multilayer or coacervate interfacial films. Layer-by-layer (LbL) deposition of oppositely charged biopolymers (e.g., proteins with anionic polysaccharides) produces thick, viscoelastic shells that resist coalescence and Ostwald ripening [66]. Similarly, pH- or ion-induced complex coacervation can generate in situ gel-like interfacial films of low permeability, slowing trans-phase mass transfer. The bigels employing gelatin–pectin coacervate coatings exhibited superior freeze–thaw and salt stability, suggesting promising applications in controlled release of nutrients and bioactives [67].

## 3. Functional Delivery Mechanisms and Potential Applications

### 3.1. Mechanistic Strategies for Bioactive Delivery

#### 3.1.1. Encapsulation and Retention Mechanism of Bioactives

Depending on molecular polarity, bioactives can be selectively entrapped within the hydrophilic hydrogel phase, the lipophilic oleogel phase, or distributed across both phases, enabling tailored stabilization and delivery performance [68]. Plant-derived hydrogels, particularly those based on polysaccharides rich in hydroxyl and carboxyl groups, are capable of forming extensive hydrogen bonding and hydrophobic interactions with polyphenols and other small-molecule bioactives [69]. These interactions enable the immobilization of functional compounds within the hydrogel’s porous matrix, reducing leaching and improving retention efficiency. Moreover, certain hydrogels are stimuli-responsive, allowing controlled release in response to environmental triggers such as pH, temperature, ionic strength, or enzymatic activity, which is particularly advantageous for targeted delivery in the gastrointestinal tract [70]. In parallel, the oleogel phase contributes to the encapsulation of lipophilic actives and supports the mechanical integrity of the overall matrix. The dual-gel system thus forms an interpenetrating network capable of co-encapsulating hydrophilic and hydrophobic bioactives with enhanced stability, higher loading capacity, and controlled release functionality compared to conventional emulsions [71]. The mechanical entrapment provided by the gel matrices also serves as a protective barrier against environmental stressors, including oxygen, UV light, and extreme pH, thereby preserving the bioactivity and shelf life of encapsulated ingredients during processing and storage [72].

Despite these advantages, achieving homogeneous distribution and high loading efficiency in biphasic systems remains challenging due to the complexity of phase homogeneity and gelation kinetics, particularly for simultaneous delivery of hydrophilic and hydrophobic bioactives. Furthermore, limited interfacial compatibility often restricts diffusion and co-delivery efficiency. Recent studies have explored hybrid formulations in which bigels were integrated with particle-based carriers or nanostructured emulsions. Such systems are regarded as advanced modifications that extend the bigel concept, enabling multi-phase structuring, enhanced encapsulation, and programmable multi-step release profiles. Rational design of bigel composition and microstructure remains essential to optimize interactions between bioactives and the matrix, thereby modulating release kinetics and improving bioavailability.

#### 3.1.2. Controlled Release: Diffusion, Degradation, and Stimuli Response

Simulated digestion models and in vitro release studies assess lipid hydrolysis and controlled nutrient delivery. Upon oral ingestion, bigels undergo a sequential transformation driven by pH gradients and enzymatic activity. In the gastric phase, low pH and gastric enzymes initiate partial swelling or degradation of the hydrogel phase, while the oleogel domain remains largely inert, protecting hydrophobic compounds from premature release [73]. As the bigels transit to the small intestine, the environment shifts to neutral pH with the presence of bile salts and pancreatic enzymes, triggering structural relaxation or erosion of the gel matrix [74]. This facilitates the site-specific release of bioactives, particularly hydrophobic compounds embedded in oleogel phase and hydrophilic molecules within the hydrogel network.

Within bigels, release kinetics are primarily governed by diffusion and matrix degradation, modulated by the structural attributes of both hydrogel and oleogel phases. The diffusion of encapsulated compounds is controlled by network porosity, crosslinking density, and the molecular affinity between bioactives and matrix [71,75]. Loosely crosslinked or highly porous hydrogels facilitate rapid diffusion, whereas dense networks impose greater steric hindrance, leading to sustained release profiles. The biphasic nature of bigels introduces additional structural complexity. In O/H system, release behavior is dictated by the biphasic architecture and interfacial dynamics described in Section 2.2. lipophilic bioactives are embedded within oleogel droplets dispersed in a continuous hydrogel matrix, where diffusion is modulated by both internal (oleogel domain) and external (hydrogel) barriers. The swelling of the hydrogel network modulates the lipase accessibility, and thus lipid hydrolysis, free fatty acid release, and diffusion of hydrophobic compounds [76]. Adjusting the hydrogel-to-oleogel ratio alters barrier thickness and tortuosity, enabling tunable release kinetics [24]. For instance, increasing hydrogel content enhanced the hydrophilic diffusion barrier and delayed curcumin release in candelilla wax oleogel-xanthan gum hydrogel-based bigels [77], while higher oleogel fractions promoted denser droplet packing and reduced permeability [78].

Beyond diffusion-mediated release, matrix degradation offers a complementary route for controlled release. Protein- or polysaccharide-based hydrogels are susceptible to enzymatic erosion under gastrointestinal conditions, enabling triggered release upon partial or complete matrix disassembly [79]. Selecting gel components with pH-sensitive or enzyme-degradable profiles (e.g., alginate, pectin, or gelatin) facilitates spatiotemporal control of release behavior [24]. Emerging strategies further incorporate stimuli-responsive functionalities into the bigel matrices to enable “smart” release. Hydrogels responsive to pH, ionic strength, or redox conditions can undergo reversible sol–gel transitions, swelling, or network relaxation to trigger release upon environmental factors [20,80,81]. A pH-sensitive gels remain collapsed in gastric acid but swell and open in neutral intestinal pH, allowing for site-specific delivery in the small intestine [71]. More advanced systems have integrated multi-responsive or self-healing features, enabling precise, sustained, or burst release in response to complex gastrointestinal stimuli.

Controlled release systems can be strategically designed to achieve both sustained and stimulus-responsive release by precisely modulating diffusion dynamics, matrix degradation rates, and environmental sensitivity. For instance, releasing a small amount of antioxidant steadily over shelf life, or a burst release of a probiotic once it reaches the colon. These controlled release strategies not only improve the efficacy of the bioactive but also help in maintaining food quality, as the active is not prematurely lost or causing off-flavors in the product.

#### 3.1.3. Targeted Delivery in Gastrointestinal Environments

**Figure 3:**
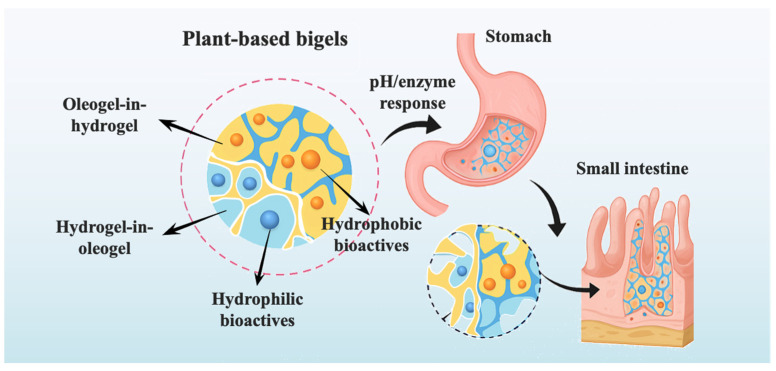
Schematic illustration of bigels during gastrointestinal digestion: pH/enzyme-triggered delivery of hydrophilic and hydrophobic bioactives in the stomach and small intestine.

Achieving targeted delivery within the gastrointestinal (GI) tract is essential for preserving the stability, viability, and bioefficacy of labile bioactives such as probiotics, enzymes, and pH-sensitive micronutrients. Plant-based bigels offer a structurally versatile biphasic matrix that protects encapsulated compounds against harsh gastric conditions while enabling site-specific release in the intestine. This targeted delivery behavior (Figure 3) builds upon the diffusion-, degradation-, and stimuli-responsive mechanisms detailed in Section 3.1.2, where phase morphology and network composition determine the release pathway. In the context of gastrointestinal (GI) delivery, plant-based bigels have been successfully designed to maintain bioactive stability through gastric conditions and enable controlled release and absorption in the intestine. Behera et al. developed a Span 40-based oleogels and polysaccharide hydrogel bigel for the co-delivery of probiotics and lipophilic bioactives, demonstrating the potential of bigels for targeted gastrointestinal release [82]. During intestinal transit, environmental cues such as neutral pH and digestive enzymes facilitated controlled hydrogel swelling and erosion, enabling the gradual release and intestinal colonization of viable probiotics. Mucoadhesive interactions enhanced retention time at the intestinal epithelium, increasing bioactive bioavailability and efficacy [83]. Further mechanistic details regarding probiotic protection and microenvironmental buffering are discussed in Section 3.2.3.

Beyond probiotics encapsulation, pH-responsive hydrogels and alginate-chitosan microbeads have been widely employed to protect peptides, enzymes, and vitamins from gastric degradation while triggering their release in the small intestine [30,84]. Recent work on alginate hydrogel-monoglyceride oleogel bigel beads demonstrated that the relatively porous alginate network not only offered robust encapsulation for hydrophilic compounds but also facilitated enhanced intestinal diffusion of epigallocatechin gallate (EGCG), resulting in accelerated release kinetics [30,84]. Moreover, modulation of the hydrogel-to-oleogel ratio has proven essential for tailoring the encapsulation efficiency and release profiles of both hydrophilic and lipophilic bioactives. Increased oleogel content decreased micellar solubilization of curcumin by decreasing emulsifier availability, thereby promoting a more sustained and controlled release [34]. Incorporating in vitro digestion models during bigel development is now standard practice to evaluate these targeting strategies. Simulated gastric and intestinal phases provide insight into structural integrity under acidic, enzyme-rich conditions, and verify that targeted release is achieved under intestinal stimuli. By achieving GI-targeted release, the bigels maximize the bioactive’s functional impact. For instance, ensuring a prebiotic fiber is mostly released in the colon, or an enzyme is released in the small intestine for absorption.

### 3.2. Functional Delivery Application of Bioactives in Plant-Based Bigels

The development of plant-based bigels as advanced delivery systems for bioactive compounds has attracted increasing interest due to the unique biphasic network, biocompatibility, and alignment with clean-label formulations. Table 1 summarizes recent progress in the encapsulation of representative bioactive classes, including polyphenols, lipophilic nutrients, and probiotics, highlighting the structural advantages and critical mechanistic insights into bigel-based delivery systems.

#### 3.2.1. Polyphenols and Flavonoids

Polyphenols and flavonoids, including curcumin, quercetin, EGCG, and anthocyanins, exhibit potent antioxidant and anti-inflammatory activities but suffer from poor solubility and chemical instability. Plant-based bigels have been shown to significantly enhance their stability, dispersibility, and bioaccessibility, enabling their integration into functional foods and nutraceutical formulations [1,34,85,86,87]. Representative work on beeswax-gelatin/carboxymethyl chitosan bigels achieved high encapsulation efficiencies of 89.7% for quercetin and 95.7% for vitamin B12, while exhibiting temperature- and pH-responsive behavior, highlighting the structural adaptability under environmental stress [88]. Similarly, gelatin-GMS bigels designed for co-delivery of curcumin and EGCG revealed distinct release kinetics that curcumin exhibited delayed gastric release followed by accelerated intestinal liberation, regulated by oleogelator concentration, whereas EGCG displayed rapid gastric release with sustained intestinal delivery [89]. These results underscore the capacity of bigel microstructure, particularly phase composition and gelator type, to precisely modulate release profiles for multiple bioactives within a single carrier matrix. Moreover, Qiu et al. developed a self-responsive color-changing bigel-based 4D printing platform, wherein curcumin and β-carotene were used as model bioactives [90]. A kinetic model quantitatively linked the observed color change to the concentration of encapsulated compounds, offering a novel visual indicator of compound release or degradation during storage. Further evidence from silk fibroin-based bigel beads co-encapsulating β-carotene and ascorbic acid demonstrated that release kinetics were governed not only by Fickian diffusion but also by hydrogel swelling, polymer relaxation, and enzymatically triggered matrix degradation [91]. These findings illustrate the importance of understanding multi-mechanistic release pathways in optimizing delivery efficacy.

#### 3.2.2. Omega-3 Fatty Acids and Lipophilic Vitamins

Omega-3 long-chain polyunsaturated fatty acids (PUFAs), such as eicosapentaenoic acid (EPA) and docosahexaenoic acid (DHA), along with lipophilic vitamins (e.g., vitamin E), are crucial for cardiovascular and neurological health but are prone to oxidative degradation and phase separation in aqueous systems. Structurally reinforced plant-based bigels provide oxidation-resistant matrices and sustained-release functionality, markedly improving nutrient retention and bioavailability in food applications. The biphasic network, composed of interpenetrating hydrogel and oleogel domains, serves as a multifunctional barrier that restricts oxygen diffusion and spatially isolates sensitive compounds, thereby mitigating oxidative deterioration [18,92]. For instance, encapsulating fish oil within bigel matrices has been shown to significantly reduce lipid peroxidation and sensory deterioration by physically entrapping the oil in a structured lipid phase while minimizing aqueous-lipid interface exposure [93,94]. Furthermore, incorporating vitamin E into the oleogel phase prior to bigel formation, further shields it from thermal and photo-oxidation while enabling targeted release along the gastrointestinal tract [95].

From a food reformulation perspective, Li et al. developed a novel bigel system composed of walnut oil-based oleogels and chitosan hydrogels, designed to partially replace conventional plastic fats. At a 75% substitution ratio, the bigel exhibited rapid melting near oral temperatures and delivered a smooth mouthfeel, indicating its potential as a functional fat alternative with health-promoting attributes [96]. Similarly, bigels formulated from coconut and olive oils have been utilized to restructure lipid distribution in food products, enabling reductions in saturated fat content while maintaining desirable rheological and textural properties [97]. In addition to enhancing oxidative stability, bigel encapsulation facilitates the incorporation of omega-3 PUFAs into aqueous food matrices, such as dairy products and beverages, by improving interfacial stabilization and promoting micellization during digestion, thus enhancing intestinal uptake.

#### 3.2.3. Probiotic Encapsulation and Viability Enhancement

Probiotic microorganisms, including *Lactobacillus* and *Bifidobacterium*, are sensitive to acidic pH, oxygen exposure, enzymatic degradation, and bile salts, resulting in significant loss of viability during food processing, storage, and digestion. Bigel-based encapsulation systems offer distinctive advantages by integrating hydrophilic and lipophilic domains that create a hierarchical barrier for protection and controlled release [83,98]. The hydrogel phase provides a hydrated, biocompatible microenvironment that buffers pH fluctuations and shields probiotic cells from pepsin-mediated degradation, while the oleogel phase acts as a secondary physical barrier limiting oxygen diffusion and moisture exchange. This dual-phase encapsulation minimizes probiotic mortality and preserves metabolic activity during gastric transit. Moreover, certain hydrogel components (e.g., sodium alginate, pectin, or carboxymethyl cellulose) exhibit intrinsic mucoadhesive properties, which enhance post-release adhesion of probiotic cells to the intestinal epithelium, thereby promoting colonization and prolonged residence time [99].

An additional dimension in the design of bigel-based probiotic systems involves the co-encapsulation of prebiotics or protective agents with the probiotics. Incorporating dietary fibers, inulin, or oligosaccharides into the hydrogel matrix can create a synbiotic environment that not only reinforces the protective matrix but also further supports the released probiotic strains, enhancing their growth and metabolic activity in situ [100,101]. Recent studies have demonstrated the practical application of bigel encapsulation in various food matrices. Yogurts, and fruit juices with bigel-encapsulated probiotics have demonstrated enhanced microbial stability and successful delivery of viable cells at therapeutically relevant levels to the lower GI tract [61,102]. These advancements highlight the potential of plant-based bigels as enabling platforms for next-generation probiotic functional foods. By integrating physical protection, mucosal adhesion, and prebiotic synergy within a single matrix, these systems support both enhanced viability and targeted delivery, ultimately amplifying the health benefits of probiotic supplementation.

**Table 1:** Applications of plant-based bigels in delivery systems for bioactives.

Delivered Target	Oleogel	Hydrogel	Oleogel/Hydrogel Ratio (*w*/*w*)	Synthesis Parameters	Emulsifier/Additives	Key Structural Features	Key Findings	Highest Release Rate (%)	Ref.
Lutein	Ethyl cellulose-sunflower oil oleogel (15% *w*/*w*)	Guar-xanthan gum hydrogel (1.5% *w*/*w*)	25:75 50:50 75:25	Oleogel at 80 °C; bigel 75 °C, 10,000 rpm, 5 min	Tween 80 (5% *w*/*w* of oil)	Hydrogel-dominant biphasic matrix with strong viscoelastic coupling	Bigels ↑ viscoelasticity & hardness; Gastric release ≈15%, intestine release up to 83%; Antioxidant activity ↑ in intestine.	83.2 (bigel 25:75)	[103]
EGCG/Curcumin	GMS-corn oil oleogel (10% *w*/*w*)	1% sodium alginate (CaCl_2_ crosslinked)	10:90 20:80 30:70	500 rpm stirring; 0.7 mm dripping; 0.5 h crosslinking	Tween 20 (0.5%)	Transitioning from a porous to a compact and continuous network (at high oleogel fractions)	Higher oleogel ↓ swelling & oil leakage; EGCG encapsulation ↓, curcumin ↑; Curcumin retained ≈70% after 40 days.	N/A	[34]
EGCG/Quercetin	N/A (double emulsion W/O/W system)	Sodium alginate (SA, 3%) hydrogel beads with soybean protein isolate	30:70 40:60 50:50	Prepare W/O/W emulsion (3:7, 4:6, 5:5), dropwise into CaCl_2_ to form beads	None	Ca^2+^-crosslinked SA forming a porous network; Double emulsions embedded within SA network	Beads ↑ encapsulation efficiency of EGCG & quercetin; Beads slowed oil digestion; EGCG bioavailability ↑ significantly.	EE: EGCG ≈ 79–84%; Q ≈ 74–80%	[104]
Curcumin	5% candelilla wax/corn oil oleogel	Potato protein hydrogel (15–25%)	70:30 90:10	1300 rpm, 10 min, blending	None	A continuous hydrogel matrix with dispersed spherical oleogel droplets	Higher protein & oleogel ↑ hardness; Curcumin bioaccessibility ↓, stability ↑; Tunable textural properties for foods.	Bioaccessibility: 16.3%; Stability: 43.8%	[105]
Curcumin	2% Span 60, almond oil	2% *w*/*v* HPMC	30:70 40:60 50:50	Oleogel at 60 °C, 500 rpm, 10 min → add hydrogel, cooling	None	Almond oil–based oleogel droplets (OG) dispersed within a continuous HPMC hydrogel (HG) matrix	Optimal BG30 (70:30) bigel: pseudoplastic, minimal oil leaching; Enhanced skin deposition & wound healing; Non-toxic, ideal for topical sustained delivery.	N/A	[106]
Catechin/Curcumin	2 wt% beeswax, algal oil	2 wt% low acyl gellan gum	20:80 40:60 50:50 60:40 80:20	Mixed with 3000 rpm for 10 min at 70 °C	None	O5H5: Bicontinuous interpenetrating structure with indistinct phase boundaries	O5H5 bicontinuous structure ↑ matrix integrity; Delayed lipolysis, CUR/CAT bioaccessibility ↑; Strong rheological–release correlation.	Bioaccessibility: curcumin 68.16%; catechin 56.16%	[6]
β-Carotene	20% monoglyceride/corn oil oleogel	1.5% κ carrageenan hydrogel	25:75 40:60 50:50 60:40 75:25	Mixed at 80 °C, cooled rapidly	None	Bicontinuous structure with interpenetrating oil and water networks; GMS crystals stabilizing droplet surfaces	Higher oleogel ↑ strength & stability; 75% oleogel ↑ light/thermal protection; 75% oleogel gave highest intestinal release.	80% in intestinal phase	[107]
Lycopene	1% GMS + 1% BW/soybean oil	0.3% high acyl gellan gum	10:90 20:80 30:70 40:60 50:50 60:40	85 °C melt → 50 °C cool, 10,000 rpm homogenize 1 min	None	O/H structure with oil droplets dispersed in a continuous gellan gum matrix; transition toward more complex or bicontinuous systems	↑ Oleogel → ↑ firmness & G′; High oleogel slowed gastric release; Thermoreversible, stable ≥3 months.	85% (10–20% oleogel)	[78]
Ascorbic acid	Ethyl cellulose-sunflower oil oleogel (15% *w*/*w*)	Xanthan guar gum hydrogel (0.75% each)	25:75 50:50 75:25	10,000 rpm, 70 °C, 5 min, rapid cooling	Tween 80 (10% *w*/*w* of oil)	Discrete irregular gel domains with lower aggregation; phase inversion from O/H to H/O above 50% oleogel	Controlled gastric release 75–87%; Higher oleogel ↑ hardness & viscoelasticity; Bigel 75:25 had highest bioaccessibility.	87 (bigel 75:25)	[108]
Coenzyme Q10	Beeswax/fish oil oleogel (10% BW)	1% Carbopol hydrogel	50:50	Oleogel: 70 °C 15 min; Bigel: 800 rpm mix	Benzalkonium chloride (preservative)	Oleogel-in-hydrogel structure combining adhesive, viscous, and lipophilic properties	Bigel ↑ permeation & flux vs. gel/oleogel; Fish oil (EPA/DHA) enhanced skin permeability; NMR/docking confirmed fatty acid-CoQ10 interaction.	0.514 mg/cm^2^ (24 h)	[93]
*L. plantarum* 299 v/Metronidazole	9% Span 40/sunflower oil	0.5% Polysaccharides (sodium alginate/CMC/maltodextrin/starch)	50:50	Organogel mixed dropwise with polysaccharide solution (50 °C) → vortex mixing → cooled to 25 °C	None	Oleogel-in-hydrogel structure with oil droplets dispersed in a continuous hydrogel phase; polysaccharide-based hydrogel network	Composition-dependent, sustained drug delivery; stable over >10 months; Branched polysaccharides enhanced *L. plantarum* viability in gastric/intestinal conditions.	Maximum viability of 10^5^–10^6^ cfu/g	[82]

Abbreviations: BW, beeswax; CMC, carboxymethyl cellulose; DHA, docosahexaenoic acid; EGCG, epigallocatechin gallate; EPA, eicosapentaenoic acid; GI, gastrointestinal; GMS, glycerol monostearate; HG, hydrogel; LA, *Lactobacillus acidophilus*; NMR, nuclear magnetic resonance; OHC, oil holding capacity; OGE, oleogel; PL, phospholipid; SUYPB, set yogurt with probiotic bigels; SWYPB, stirred yogurt with probiotic bigels; WPC/WPC80, whey protein concentrate (80% protein). Symbols: ↑ indicates an increase; ↓ indicates a decrease; “N/A” indicates that the item was not detected or not applicable; and “→” indicates continuity between steps.

## 4. Challenges and Future Perspectives

### 4.1. Current Challenges for Plant-Based Bigels in Functional Delivery

Despite notable progress in the development of plant-based bigels for functional delivery, several critical challenges hinder the industrial translation and broad application of plant-based bigels in foods.

(i) Scale-up and manufacturability. Production typically requires sequential preparation of oleogel and hydrogel phases followed by precise integration under controlled shear, temperature, and gelation kinetics. Preserving droplet size distributions, phase continuity, and asepsis at scale, especially for heat- and shear-labile actives (e.g., probiotic cells, enzymes), is technically demanding. Moreover, limited compatibility with continuous processing equipment constrains adoption on conventional lines and complicates process validation.

(ii) Raw-material variability and design predictability. Variability in raw material quality and processing parameters also leads to inconsistent product performance. Minor fluctuations in the molecular weight of biopolymers, oil composition, or cooling rates can significantly alter rheological properties, release kinetics, and sensory characteristics. This underlines the urgent need for predictive structure-function relationships and quantitative models that can guide the rational design, optimization, and quality control of bigel-based systems.

(iii) Regulatory and clean-label constraints. All components, including gelators, emulsifiers, crosslinkers, and processing aids, must be food-grade and approved by relevant authorities. Reliance on Generally Recognized As Safe (GRAS) plant-derived polymers (e.g., starches, pectins, alginates, plant proteins) supports clean-label positioning but typically offer lower mechanical strength, thermal stability, or pH tolerance compared to synthetic analogs, limiting the functional robustness of the final bigel product. In some cases, regulatory limitations on usage levels may conflict with concentrations required to achieve desirable rheological and release characteristics.

### 4.2. Future Perspectives

To fully realize the potential of plant-based bigels as multifunctional delivery platforms, future research should focus on three interrelated objectives: (i) designing structurally robust and multi-stimuli-responsive networks using GRAS-compliant biopolymers; (ii) developing scalable and economically viable processing technologies; and (iii) integrating multiscale modeling to predict and optimize behavior from formulation through gastrointestinal transit.

One promising direction is the development of multi-stimuli bigels capable of sequential or synergistic responses, such as pH-triggered swelling followed by enzyme-mediated degradation. Incorporating novel external stimuli, including magnetic fields, light, or mild electric triggers, could further enable programmable, on-demand release of encapsulated bioactives. Realizing this will require innovations in gelation chemistry, such as cleavable crosslinkers, dynamic covalent bonding (e.g., Schiff base linkages, disulfide bridges), and semi-interpenetrating polymer networks with tunable degradation kinetics. Moreover, integrating computational modeling and molecular dynamics simulations offers a pathway to quantitatively predict diffusion, network relaxation, and release kinetics, supporting the rational design of bigels with precisely controlled delivery profiles. These innovations represent the next stage of intelligent food-grade delivery systems that combine adaptability, selectivity, and predictive tunability.

Cross-disciplinary integration with pharmaceutical and biomaterials science holds substantial potential. Techniques such as microfluidics, 3D bioprinting, and nanoencapsulation, well-established in drug delivery, can be adapted to fabricate bigels with precisely engineered microstructures and controlled release dynamics. Conversely, plant-based bigels offer biocompatible, biodegradable, and edible alternatives for biomedical applications, including oral or mucosal delivery of vaccines, enzymes, and therapeutic peptides. This cross-pollination of technologies may increasingly blur the boundary between functional foods and nutraceuticals, ultimately leading to edible platforms with clinical-grade delivery precision.

## 5. Conclusions

This review comprehensively explores the recent progress in the structural design, stabilization strategies, and functional delivery performance of plant-based bigels. By integrating plant-derived hydrocolloids and oleogelators within a unified biphasic matrix, bigels offer a promising clean-label and sustainable alternative for functional foods. Their tunable viscoelastic properties, responsive interfacial structures, and biphasic diffusion dynamics facilitate enhanced stability, bioaccessibility, and site-specific release of vulnerable nutraceuticals such as polyphenols, omega-3 fatty acids, probiotics, and fat-soluble vitamins. However, critical challenges remain, particularly in preserving structural integrity under industrial processing conditions, scaling up complex multiphase systems while maintaining product reproducibility, and navigating formulation within the constraints of food-grade regulatory frameworks. Future research should prioritize the development of stimuli-responsive smart bigels, capable of site-specific or time-controlled release. Parallelly, the integration of computational modeling, machine learning, and high-throughput screening can further accelerate rational formulation and predict structure–function relationships. Additionally, sustainability-focused innovations, such as the valorization of agri-food by-products as gel matrices and the utilization of naturally derived oleogelators, will be instrumental in advancing circular bioeconomy goals.

## Data Availability

No new data were created or analyzed in this study. Data sharing is not applicable to this article.

## Abbreviations

The following abbreviations are used in this manuscript:

LMWGs	Low-molecular-weight gelators
HMWGs	High-molecular-weight gelators
EC	Ethyl cellulose
GMS	Glycerol monostearate
O/H	Oleogel-in-hydrogel
SPI	Soy protein isolate
CNCs	Cellulose nanocrystals
GI	Gastrointestinal
EGCG	Epigallocatechin gallate
PUFAs	Polyunsaturated fatty acids
EPA	Eicosapentaenoic acid
DHA	Docosahexaenoic acid
GRAS	Generally Recognized As Safe
